# Exploring Protein Binding of Uremic Toxins in Patients with Different Stages of Chronic Kidney Disease and during Hemodialysis

**DOI:** 10.3390/toxins7103933

**Published:** 2015-09-28

**Authors:** Olivier Deltombe, Wim Van Biesen, Griet Glorieux, Ziad Massy, Annemieke Dhondt, Sunny Eloot

**Affiliations:** 1 Department of Internal Medicine, Nephrology Section, Ghent University Hospital, Ghent 9000, Belgium; E-Mails: wim.vanbiesen@ugent.be (W.V.B.); griet.glorieux@ugent.be (G.G.); annemie.dhondt@ugent.be (A.D.); sunny.eloot@ugent.be (S.E.); 2 Division of Nephrology, Amiens University Hospital, Amiens 80000, France; E-Mail: massy@u-picardie.fr

**Keywords:** chronic kidney disease, hemodialysis, protein binding, uremic toxins, *p*-cresylglucuronide, hippuric acid, indole-3-acetic acid, indoxyl sulfate, *p*-cresylsulfate

## Abstract

As protein binding of uremic toxins is not well understood, neither in chronic kidney disease (CKD) progression, nor during a hemodialysis (HD) session, we studied protein binding in two cross-sectional studies. Ninety-five CKD 2 to 5 patients and ten stable hemodialysis patients were included. Blood samples were taken either during the routine ambulatory visit (CKD patients) or from blood inlet and outlet line during dialysis (HD patients). Total (C_T_) and free concentrations were determined of *p*-cresylglucuronide (*p*CG), hippuric acid (HA), indole-3-acetic acid (IAA), indoxyl sulfate (IS) and *p*-cresylsulfate (*p*CS), and their percentage protein binding (%PB) was calculated. In CKD patients, %PB/C_T_ resulted in a positive correlation (all *p* < 0.001) with renal function for all five uremic toxins. In HD patients, %PB was increased after 120 min of dialysis for HA and at the dialysis end for the stronger (IAA) and the highly-bound (IS and *p*CS) solutes. During one passage through the dialyzer at 120 min, %PB was increased for HA (borderline), IAA, IS and *p*CS. These findings explain why protein-bound solutes are difficult to remove by dialysis: a combination of the fact that (i) only the free fraction can pass the filter and (ii) the equilibrium, as it was pre-dialysis, cannot be restored during the dialysis session, as it is continuously disturbed.

## 1. Introduction

Uremic syndrome is characterized by the retention of a large number of compounds, which in healthy persons are excreted by the kidneys. Some of those retention solutes interact negatively with biological functions and are called uremic toxins. These toxins are classified into three groups: the free small water-soluble solutes (molecular weight (MW) < 500 Da), the middle molecules (MW > 500 Da) and the protein-bound solutes [1,2]. The latter solutes have a protein binding ranging from around 10% (e.g., *p*-cresylglucuronide) to near 100% (e.g., *p*-cresylsulfate). Many of these protein-bound substances are known to exert toxicity in a direct or indirect way [3,4,5]. However, in our current understanding, toxicity is only exerted by the free concentration and not by the protein-bound concentration.

Since the extent of protein binding depends on the solute concentration, the protein concentration, the protein-solute affinity constant and the presence or absence of competing solutes [6], each protein-bound uremic toxin binds to the specific proteins to a variable degree. Structural changes of proteins can also alter the extent of protein binding, and therefore, it can be hypothesized that the degree of protein binding also changes in individual patients with the progression of their chronic kidney disease (CKD), as post-translational modifications (oxidation, carbamylation and glycosylation as the most relevant processes) of proteins increase as CKD progresses [7]. During hemodialysis (HD), only the free fraction can be removed, such that the overall dialyzer clearance depends on the free toxin concentration and on the speed of equilibration between bound and free fractions. Free fractions, and with it dialyzer clearance, can be increased, for example, by the presence of competing ligands in the serum, like sodium octanoate [8,9], or by infusing hypertonic saline at the dialyzer inlet [10,11,12]. The mechanisms of protein binding are, however, not well understood. 

In this study, we evaluated the percentage protein binding at different stages of CKD (*i.e.*, Stages 2 to 5), during a hemodialysis session in dialysis patients and in healthy controls. This information might be useful in the development of new removal strategies aiming at the optimization of dialysis.

## 2. Results and Discussion

### 2.1. Patient Characteristics

Table 1, Table 2 and Table 3 present the demographic and clinical characteristics of the 95 CKD patients, the 10 HD patients and the 10 healthy controls with normal renal function, respectively. Besides differences in renal function among the CKD stages, no dissimilarities were observed among the different CKD stages for age, gender, body mass index (BMI), diabetes mellitus (DM) and albumin concentration.

**Table 1:** Demographic and clinical characteristics of the CKD patients.

Characteristics	CKD patients				
	CKD 2 to 5	CKD 2	CKD 3	CKD 4	CKD 5
**Number, *n* (%)**	95 (100)	11 (11.5)	37 (39)	37 (39)	10 (10.5)
**Age (years)**	69 (59;76)	62 (59;71)	74 (61;77)	69 (55;74)	79 (60;83)
**Male gender, *n* (%)**	59 (62)	9 (82)	24 (65)	22 (60)	4 (40)
**BMI (kg/m^2^)**	29 (25;32)	27 (21;29)	29 (25;32)	29 (26;34)	25 (23;30)
**DM, *n* (%)**	45 (47)	4 (36)	19 (51)	18 (49)	4 (40)
**Albumin (g/L)**	39 (35;44)	42 (37;47)	38 (35;42)	41 (35;44)	33 (28;39)
**Renal function ^1^ (mL/min)**	32 (20;49)	67 (63;71)	45 (35;51)	22 (19;25) °^,+^	11 (9;13) °^,+^

CKD: chronic kidney disease; BMI: body mass index; DM: diabetes mellitus. Median (25th percentile (pct); 75th pct). ° *p* < 0.05 *versus* CKD 2; ^+^ *p* < 0.05 *versus* CKD 3. ^1^ Renal function calculated according to the Cockcroft-Gault formula.

**Table 2:** Demographic and clinical characteristics of the HD patients.

Characteristics	HD patients
**Age (years)**	72 (61;78)
**Male gender, *n* (%)**	8 (80)
**Ultrafiltration (mL/min)**	4.8 (3.5;8.9)
**BMI (kg/m^2^)**	28 (25;28)
**DM, *n* (%)**	5 (50)
**Total protein (g/L)**	60 (58;67)
**Renal function (mL/min)**	2.6 (0.0;4.1)

HD: hemodialysis. Median (25th pct; 75th pct).

**Table 3:** Demographic and clinical characteristics of the healthy controls.

Characteristics	Healthy controls
**Age (years)**	40 (33;57)
**Male gender, *n* (%)**	4 (40)
**BMI (kg/m^2^)**	23 (19;27)

Median (25th pct; 75th pct).

The median percentage protein binding (%PB), the free and total concentration of *p-*cresylglucuronide (*p*CG), hippuric acid (HA), indole-3-acetic acid (IAA), indoxyl sulfate (IS) and *p-*cresylsulfate (*p*CS) are shown in Table 4 for an increasing degree of kidney failure, *i.e.*, consecutively for the healthy controls, the CKD patients and the HD patients. Since the three groups differ in origin, nutrition and hospital center, they were not statistically compared to each other.

For the healthy controls, the free and total concentration of *p*CG were below the limit of quantification (LOQ), and the %PB could not be calculated. The median %PB was 34% (HA), 83% (IAA), 84% (IS) and 94% (*p*CS).

**Table 4:** Percentage protein binding (%PB), free (C_F_) and total (C_T_) concentration of protein-bound solutes in healthy controls with normal renal function, in CKD and HD patients (pre-dialysis).

Uremic Toxin	%PB or Concentration	Healthy controls	CKD				HD
			CKD 2	CKD 3	CKD 4	CKD 5	
***p*CG**	*%PB C_F_ (mg/dL) C_T_ (mg/dL)*	- <LOQ <LOQ	7(3;24) 0.03(0.03;0.04) 0.03(0.03;0.05)	10(6;14) 0.03(0.03;0.04) 0.04(0.03;0.04)	9(6;16) 0.04(0.03;0.07) 0.05(0.03;0.08)	8(5;20) 0.20(0.07;0.25) °^,+^ 0.25(0.08;0.28) °^,+,#^	12(9;19) 0.31(0.22;0.85) 0.35(0.25;0.99)
**HA**	*%PB C_F_ (mg/dL) C_T_ (mg/dL)*	34(22;39) 0.13(0.10;0.16) 0.17(0.11;0.25)	38(34;42) 0.23(0.18;0.38) 0.39(0.32;0.58)	38(34;43) 0.20(0.18;0.32) 0.33(0.27;0.53)	38(35;44) 0.30(0.21;0.42) 0.51(0.33;0.53)	43(36;45) 0.54(0.34;0.69) ^+^ 0.93(0.51;1.25) ^+^	39(32;54) 1.59(0.73;3.30) 2.41(1.57;5.42)
**IAA**	*%PB C_F_ (mg/dL) C_T_ (mg/dL)*	83(78;84) 0.01(0.01;0.01) 0.04(0.03;0.05)	60(56;66) 0.02(0.02;0.02) 0.06(0.05;0.07)	67(61;75) 0.03(0.02;0.03) 0.08(0.06;0.12)	66(61;72) 0.03(0.03;0.03) °^,(+)^ 0.09(0.07;0.11) °	68(65;71) 0.03(0.03;0.04) °^,+^ 0.11(0.10;0.15) °	69(63;80) 0.07(0.04;0.11) 0.19(0.13;0.33)
**IS**	*%PB C_F_ (mg/dL)C_T_ (mg/dL)*	84(77;88) 0.02(0.01;0.02) 0.10(0.06;0.14)	77(71;83) 0.03(0.03;0.03) 0.16(0.11;0.18)	86(80;90) 0.03(0.03;0.03) 0.23(0.16;0.35)	89(87;92) °^,+^ 0.04(0.03;0.04) °^,+^ 0.36(0.28;0.55) °^,+^	92(90;95) °^,+^ 0.06(0.03;0.08) °^,+^ 0.79(0.31;1.50) °^,+^	93(90;95) 0.08(0.04;0.21) 1.40(0.69;2.18)
***p*CS**	*%PB C_F_ (mg/dL) C_T_ (mg/dL)*	94(87;96) 0.02(0.01;0.02) 0.31(0.08;0.47)	93(89;96) 0.05(0.03;0.07) 0.47(0.38;0.70)	97(96;97) ° 0.04(0.02;0.05) 0.95(0.59;1.37)	96(95;97) 0.05(0.03;0.12) 1.19(0.65;2.52) °	94(93;95) ^+^ 0.21(0.12;0.31) ^+,#^ 3.29(1.52;4.47) °^,+^	95(93;97) 0.06(0.04;0.10) 2.06(1.14;2.87)

*p*CG: *p*-cresylglucuronide; HA: hippuric acid; IAA: indole-3-acetic acid; IS: indoxyl sulfate; *p*CS: *p*-cresylsulfate. LOQ: limit of quantification; Median (25th pct; 75th pct). ° *p* < 0.05 *versus* CKD 2; ^+^ *p* < 0.05 *versus* CKD 3; ^#^ *p* < 0.05 *versus* CKD 4; (+) *p* = 0.061.

In the different stages of CKD, the free and total concentrations of the weakly-bound solutes *p*CG and HA were only increased in CKD 5 patients, and for the stronger bound solutes IAA, IS and *p*CS, already at CKD Stage 4. The median percentage protein binding was in the range 7% to 8% (*p*CG), 38% to 43% (HA), 60% to 68% (IAA), 77% to 92% (IS) and 93% to 94% (*p*CS). For the highly-bound IS, %PB was increased in CKD 4 and 5 patients with respect to CKD 2 and 3 patients, while for *p*CS, %PB showed some variation, but without a clear trend. The median %PB in the HD patients was 12% (*p*CG), 39% (HA), 69% (IAA), 93% (IS) and 95% (*p*CS).

### 2.2. CKD Patients

**Figure 1:**
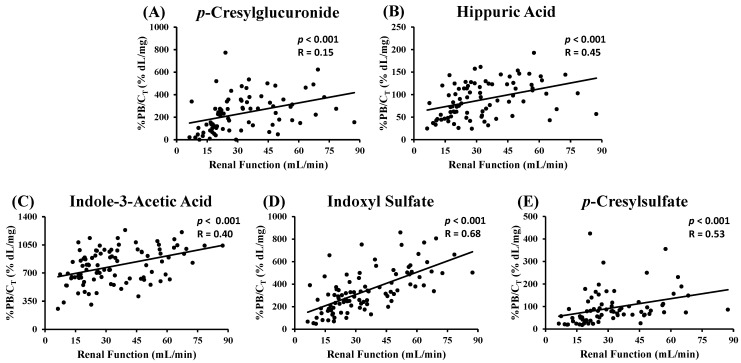
Percentage protein binding (%PB) normalized for total toxin concentration (C_T_) *versus* renal function of CKD patients for: (**A**) *p*-cresylglucuronide; (**B**) hippuric acid; (**C**) indole-3-acetic acid; (**D**) indoxyl sulfate; and (**E**) *p*-cresylsulfate.

Considering the 95 CKD patients, only %PB of IS showed an (inverse) correlation with renal function (*R* = −0.64; *p* < 0.001) (Figure S1 in the Supplementary Materials and Table 5). Normalizing %PB for total toxin concentration, however, resulted in a positive correlation (all *p* < 0.001) with renal function for all five uremic toxins (Figure 1).

Table 5 shows the *p*- and *R*^2^-values of the correlation test between %PB and renal function in the 95 CKD patients. The influence of added covariates C_T_, diabetes mellitus and the albumin concentration was checked, as shown in Table 5.

The total toxin concentration improved the model (all *p* < 0.05), except for *p*CG (Table 5). This result is in line with the correlations found in Figure 1.

When diabetes mellitus was added to the model, only an improvement was found for IAA and IS (Table 5). However, a lower *R*^2^-value was given compared to C_T_.

Including albumin concentration did not improve the correlation between %PB and renal function (Table 5), neither did it correlate with %PB (Figure S2). Therefore, the albumin concentration (in this range) did not have an influence on the %PB, as already published for other compounds in the literature [13,14].

**Table 5:** *p*- and *R*^2^-values of the correlations between %PB and renal function for *p*CG, HA, IAA, IS and *p*CS and the influence of added covariates total toxin concentration (C_T_), diabetes mellitus (DM) and albumin concentration in CKD patients.

Uremic Toxin	%PB *versus* RF		Covariates					
			C_T_		DM		Albumin	
	*p*	*R*^2^	*p*	*R*^2^	*p*	*R*^2^	*p*	*R*^2^
***p*CG**	0.65	-	0.081	-	0.288	-	0.91	-
**HA**	0.40	-	0.008	0.23	0.623	-	0.49	-
**IAA**	0.18	-	0.002	0.71	0.028	0.034	0.85	-
**IS**	<0.001	0.41	<0.001	0.67	<0.001	0.317	0.77	-
***p*CS**	0.12	-	0.004	0.16	0.843	-	0.62	-

*p* < 0.05 is indicated in bold. *R*^2^-values only shown in the case of significant *p*.

It is known that post-translational modifications of proteins increase as CKD progresses, with carbamylation, oxidation, glycosylation and guanidinylation as the most relevant processes [7,15]. Whether our result is influenced by these structural changes in the proteins or whether this is the result of competitive binding, leading to an enhanced free toxin concentration, remains unclear. Anyway, the presented results are in line with those for highly (around 90%) protein-bound compounds, like valproic acid or phenytoin, also showing a decreased protein binding in patients with renal failure [16,17].

### 2.3. HD Patients

**Figure 2:**
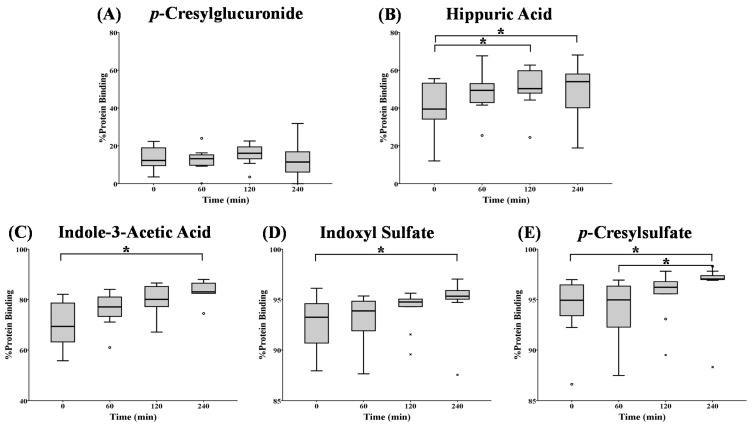
Percentage protein binding at different time points during a hemodialysis (HD) session for: (**A**) *p*-cresylglucuronide; (**B**) hippuric acid; (**C**) indole-3-acetic acid; (**D**) indoxyl sulfate; and (**E**) *p*-cresylsulfate. * *p* < 0.05; o: outlier; x: extreme.

In hemodialysis patients, median pre-dialysis %PB was 12% (*p*CG), 39% (HA), 69% (IAA), 93% (IS) and 95% (*p*CS). Figure 2 and Table 6 show %PB determined at the inlet of the dialyzer, at the start (0 min) of the hemodialysis session and after 60, 120 and 240 min.

The total and free toxin concentrations were decreased from 120 min on for *p*CG, HA and *p*CS. For IAA, only the total concentration decreased from 120 min on and for IS, only the free concentration (Table 6). No differences in %PB were observed during the HD session for the weakly-bound *p*CG. For HA, %PB was increased after 120 and 240 min *versus* HD start, while for the stronger (IAA) and highly-bound (IS and *p*CS) solutes, this increase was only significant after 240 min (Figure 2 and Table 6).

**Figure 3:**
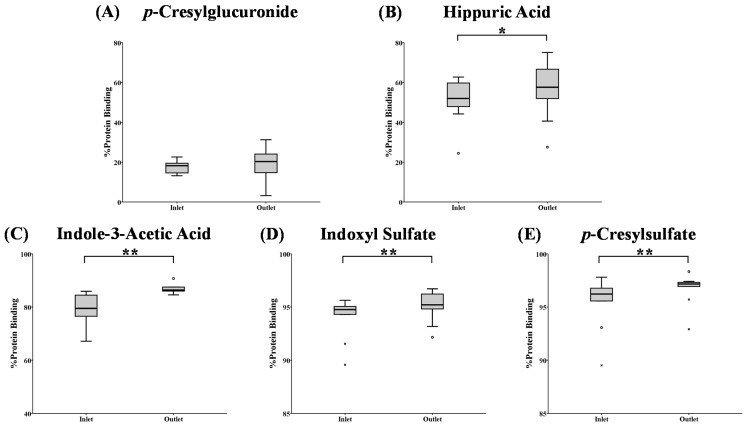
Percentage protein binding at the dialyzer inlet *versus* outlet after 120 min since dialysis start for: (**A**) *p*-cresylglucuronide; (**B**) hippuric acid; (**C**) indole-3-acetic acid; (**D**) indoxyl sulfate; and (**E**) *p*-cresylsulfate. ** *p* < 0.05 *versus* the inlet; * *p* = 0.066 *versus* the inlet; o: outlier; x: extreme.

Changes in percentage protein binding from the dialyzer inlet towards the outlet as measured at 120 min after dialysis start are depicted in Figure 3. For the weakly-bound *p*CG, passage through the dialyzer did not influence the percentage binding. For HA, the %PB was increased with a borderline significance (*p* = 0.066). The percentage protein binding for the stronger (IAA) and the highly-bound (IS and *p*CS) solutes was significantly increased from dialyzer inlet to outlet at 120 min. 

**Table 6:** Percentage protein binding, free and total concentration of protein-bound solutes in HD patients at different time points during an HD session.

Uremic Toxin	%PB or Concentration	0 min	60 min	120 min	240 min
***p*CG**	*%PB C_F_ (mg/dL) C_T_ (mg/dL)*	12(9;19) 0.31(0.22;0.85) 0.35(0.25;0.99)	13(10;15) 0.14(0.11;0.44) 0.17(0.12;0.45)	10(13;20) 0.11(0.07;0.29) ° 0.14(0.08;0.36) °	11(5;19) 0.08(0.04;0.16) °^,+^ 0.10(0.05;0.19) °^,+^
**HA**	*%PB C_F_ (mg/dL) C_T_ (mg/dL)*	39(32;54) 1.59(0.73;3.30) 2.41(1.57;5.42)	49(43;54) 0.91(0.41;1.84) 1.42(0.99;3.52)	50(47;60) ° 0.70(0.44;1.37) °1.37(0.97;2.55) °	54(39;58) ° 0.42(0.32;0.82) °^,+^ 0.95(0.61;1.49) °^,+^
**IAA**	*%PB C_F_ (mg/dL) C_T_ (mg/dL)*	69(63;80) 0.07(0.04;0.11) 0.19(0.13;0.33)	77(72;82) 0.04(0.02;0.08) 0.14(0.10;0.26)	80(77;86) 0.03(0.02;0.06) 0.13(0.10;0.23) °	83(78;87) °0.03(0.02;0.07) ° 0.10(0.08;0.16) °^,+^
**IS**	*%PB C_F_ (mg/dL) C_T_ (mg/dL)*	93(90;95) 0.08(0.04;0.21) 1.40(0.69;2.18)	94(91;95) 0.06(0.04;0.16) 1.14(0.60;1.97)	95(94;95) 0.06(0.03;0.12) ° 1.06(0.56;1.75)	95(95;96) ° 0.03(0.02;0.07) °^,+^ 0.72(0.48;1.47) °^,+^
***p*CS**	*%PB C_F_ (mg/dL) C_T_ (mg/dL)*	95(93;97) 0.12(0.10;0.24) 2.76(1.75;4.25)	95(92;96) 0.13(0.09;0.19) 2.46(1.45;3.70)	96(95;97) 0.09(0.07;0.14) ° 2.27(1.32;3.41) °	97(97;97) °^,+^ 0.06(0.04;0.10) °^,+^ 2.06(1.14;2.87) °^,+^

° *p* < 0.05 *versus* 0 min; ^+^ *p* < 0.05 *versus* 60 min. Median (25th pct; 75th pct).

This is the first time that it has been demonstrated that the percentage protein binding for stronger bound solutes changes during dialysis, and this is both within the dialyzer itself during a single passage and within a patient (*i.e.*, during the course of the dialysis session). The influence of hemoconcentration on these observations was checked by a correlation test between the change in %PB and the change in total protein concentration at the inlet and outlet of the dialyzer, but no significant correlation was found (Figure S3). Neither can this be explained by changes in pH, since we only observed limited pH changes during the course of a dialysis session. 

**Figure 4:**
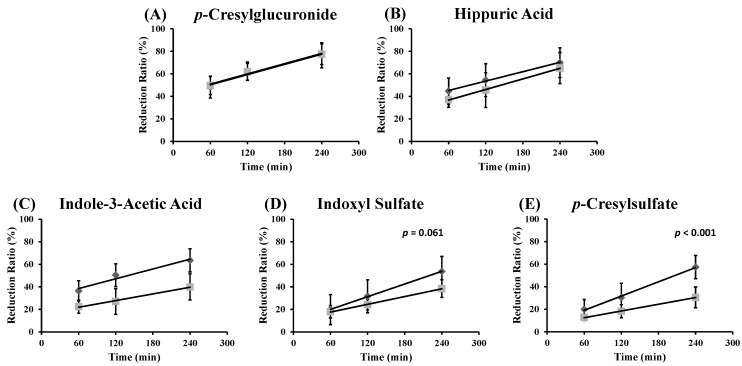
Reduction ratio for total (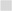) and free (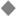) toxin concentration at different time points during an HD session for: (**A**) *p*-cresylglucuronide (free: y = 0.15x + 41; *R*^2^ = 0.96; total: y = 0.15x + 42; *R*^2^ = 0.99); (**B**) hippuric acid (free: y = 0.14x + 37; *R*^2^ = 1.00; total: y = 0.16x + 27; *R*^2^ = 1.00); (**C**) indole-3-acetic acid (free: y = 0.14x + 30; *R*^2^ = 0.96; total: y = 0.10x + 16; *R*^2^ = 0.99); (**D**) indoxyl sulfate (free: y = 0.19x + 8.7; *R*^2^ = 1.00; total: y = 0.11x + 11; *R*^2^ = 1.00); and (**E**) *p*-cresylsulfate (free: y = 0.21x + 6.4; *R*^2^ = 1.00; total: y = 0.10x + 6.5; *R*^2^ = 1.00).

A possible explanation might be found in the hypothesis that the physicochemical bond of the toxin with its protein is strong. As a consequence, the equilibrium reaction is too slow to restore the free toxin concentration within the time frame of a single passage through the dialyzer and even within the time frame of a dialysis session once the pool of available pre-dialysis free fraction has been removed. Therefore, we calculated the reduction ratio (RR) for both total and free toxin concentration to check this hypothesis. The RR is graphically presented in Figure 4 for *p*CG, HA, IAA, IS and *p*CS at 60, 120 and 240 min since the start of the dialysis session. The slopes of free and total RR are borderline significant for IS (*p* = 0.061) and significantly different for *p*CS (*p* < 0.001). 

The difference in RR between free and total concentrations might imply that the equilibrium could not be formed during the course of the dialysis session. For the weakly-bound *p*CG, for example, around 90% of the total concentration is unbound and is thus easily removed by the dialyzer, resulting in a comparable total and free reduction ratio. The RR of the highly-bound *p*CS, on the other hand, is different for free and total concentrations, and within the four hours of dialysis, the RR of the total concentration cannot follow the RR of the free concentration (around 4%). The equilibrium between free and bound *p*CS is continuously disturbed because of the dynamic process of dialysis. Therefore, the equilibrium (as it was pre-dialysis) cannot be restored during the dialysis session and explains the observations in Figure 2 and Figure 3.

Both aspects, *i.e.*, (i) only the free fraction can be removed and (ii) the bound fraction is released slowly, are the main determinants for the limited removal of solutes during dialysis. This fits with the multi-pass device observations, as described by Eloot *et al.*, where removal of protein-bound solutes was limited to the first two hours of dialysis [18]. In a kinetic modelling study from our group [19] based on intradialytic concentrations, we found an inverse correlation between dialyzer clearance and the %PB. Furthermore, total distribution volumes and intercompartment clearances (except for *p*CG), which are representative for solute retardation inside the patients, were also inversely correlated with the %PB. Thus, the present findings are in full agreement with those in the kinetic analysis. 

It can be stated that during dialysis, first the free fraction will be removed and will cause a disequilibrium with the bound fraction, as well as with concentrations in the extra vascular spaces. This results in a continuous release of the bound fraction, respectively inflow from the extravascular space. These kinetics of protein-bound solutes were already extensively studied by our group showing the multi-compartmental behavior with vascular and extravascular spaces [20]. 

Enhancing the filtration of proteins is cumbersome, as this would result in hypoproteinemia. However, it can be hoped that strategies could be developed that change the strength of the physical bond between the toxin and its ligand, to increase the free (dialyzable) solute concentration.

Recent research suggested a novel approach to increase the free fraction of the protein-bound solutes phenyl acetic acid (PAA), indoxyl sulfate and *p-*cresylsulfate during dialysis by infusing a hypertonic solution at the dialyzer inlet [10,11,12]. This increased the local ionic strength at the blood inlet of the dialyzer, resulting in an enhanced release of uremic toxin from its protein binding site, most pronounced for the middle bound PAA (%PB around 60%). With this approach, the clearance during *in vitro* dialysis was relatively most beneficial for the highly-bound IS and *p*CS [10]. These *in vitro* results might be promising, but the absence of hemolysis due to hyperosmolarity needs to be further investigated *in vivo*.

## 3. Experimental Section

To calculate the percentage protein binding in CKD patients, data were taken from a cross-sectional study including 95 patients with confirmed diagnosis of CKD Stages 2 to 5. To unravel changes in protein binding in the hemodialyzer, as well as during the course of a hemodialysis session, data were taken from a second cross-sectional study including 10 stable HD patients. Data from ten healthy controls with normal renal function were also collected to cover the range (healthy-CKD-HD) of free and total toxin concentrations, as well as for protein binding.

### 3.1. Patients and Sampling Protocol

CKD patients: 95 CKD patients (CKD Stages 2 to 5) were included from Amiens University Hospital (France), in whom concentrations of uremic retention solutes were evaluated for their relation to clinical outcomes [21,22,23]. These patients were older than 40 years and had a confirmed diagnosis of CKD (renal function below 90 mL/min, calculated in the aforementioned study according to the Cockcroft-Gault formula [24]). Exclusion criteria included the presence of chronic inflammatory disease, atrial fibrillation, complete heart block, abdominal aorta aneurysm, aortic and/or femoral artery prosthesis, primary hyperparathyroidism, kidney transplantation, on dialysis and any acute cardiovascular event in the 3 months before screening for inclusion. Blood samples were taken in the morning on the occasion of a visit at the outpatient clinic. 

HD patients: 10 stable hemodialysis patients were included from Ghent University Hospital. Exclusion criteria were active infection, pregnancy, unstable condition, vascular access problems and age below 18 years. During the experimental session at midweek, conventional two needle/lumen HD was performed for 240 min using high-flux dialyzers: FX800 (*n* = 6) (Fresenius Medical Care, Bad Homburg, Germany), Evodial (*n* = 1) (Gambro, Lund, Sweden), Xenium 210 (*n* = 1) (Baxter, Dearfield, IL, USA), Phylter HF17G (*n* = 1) and Phylter HF17SD (*n* = 1) (Bellco, Mirandola, Italy) in a diffusive mode. Blood and dialysate flows were set at 300 and 700 mL/min, respectively, while ultrafiltration rates were set according to the needs of the patients. Nine patients had a well-functioning fistula and one patient a Bard Optiflow central venous catheter (Bard, Covengton, GA, USA) as vascular access. Residual renal function was calculated as the arithmetic mean of the creatinine and urea clearance, calculated from the interdialytic urine collection (volume and concentration) and blood concentrations at the start and end of the interdialytic period [25]. During the experimental session, blood samples were collected at the start of the session from the vascular access and from the inlet blood line after 60 and 120 min, and immediately after discontinuation of the dialysis session (at 240 min). Blood samples were also collected from the outlet blood line after 120 min since the start of the dialysis session.

Both studies were approved by the local ethical committees (Comité de protection des Personnes Nord-Ouest II, CHU Amiens, Amiens, France, 06H3 for CKD patients, and Ghent University Hospital, Ghent, Belgium, UZG 2008/081 for HD patients), performed in accordance with the principles of the Declaration of Helsinki, and all patients gave their written informed consent.

Healthy Controls: Data from 10 healthy volunteers with normal renal function were collected at Ghent University Hospital. Subjects who were smoking, had an infection, were pregnant or on medication were excluded.

### 3.2. Laboratory

All blood samples were immediately centrifuged after sampling, and serum was stored at −80 °C until batch analysis.

Concentrations of protein-bound uremic toxins were determined by reversed-phase high performance liquid chromatography (RP-HPLC), as described earlier [26,27]. The solutes analyzed were *p*-cresylglucuronide (*p*CG, MW: 284.3 Da), hippuric acid (HA, MW: 179.2 Da), indole-3-acetic acid (IAA, MW: 174.2 Da), indoxyl sulfate (IS, MW: 212.2 Da) and *p*-cresylsulfate (*p*CS, MW: 187.2 Da). To determine the total concentration, serum samples were first deproteinized by heat denaturation prior to HPLC analysis [27]. HA was analyzed by UV detection at 254 nm, whereas *p*CG and *p*CS (λ_exc._ = 265 nm, λ_em_ = 290 nm) and IAA and IS (λ_exc._ = 280 nm, λ_em_ = 340 nm) were determined by fluorescence detection [26,27]. To obtain free fractions, untreated serum samples were filtered through a Centrifree^®^ filter device (Millipore Billerica, MA, USA) prior to heating [26]. Albumin levels in serum from CKD patients were assayed in a biochemistry laboratory using standard autoanalyzer techniques (the Modular IIP system, Roche Diagnostics, Basel, Switzerland) [21,22,23]. In HD patients, total protein concentration in serum was analyzed according to standard methods (Biuret reaction).

### 3.3. Calculations

Percentage protein binding (%PB) was calculated from the measured total (C_T_) and free (C_F_) concentrations as %PB = [1 − (C_F_/C_T_)] × 100%.

The reduction ratio (RR) for the free and total concentration in HD patients was determined from the concentration at the start of the dialysis session (C_pre_) and after time *t* = 60, 120 and 240 min (C_t_) as RR = [C_pre_ − (C_t_/C_pre_)] × 100%. 

### 3.4. Statistical Analysis

Statistical evaluation was performed with SPSS Statistics 22 (2013, Armonk, NY, USA). Data were checked for normality. As most numeric data were not normally distributed, data were expressed as the median (25th percentile (pct); 75th pct). To compare independent categorical data, Fisher’s exact test was performed. Differences between more than two groups of unpaired data (CKD data) were checked with a Kruskal–Wallis test (with multiple comparisons and Bonferroni correction). Paired comparisons (HD data) between more than two groups were made with a Friedman test (with multiple comparisons and Bonferroni correction). To evaluate the difference between two paired groups, the Wilcoxon signed-rank test was applied. Spearman’s rho test was performed to check correlation (all presented *p*- and *R*-values are Spearman’s rho values, unless stated otherwise). A univariate general linear model (GLM) was used with covariates (C_T_, DM and albumin concentration) to check any improvements of the correlation between renal function and %PB. A linear regression procedure was used to check differences in regression coefficients between two groups (reduction ratio). *p* < 0.05 was considered significant, and all tests were two-tailed.

## 4. Conclusions

In this study, we explored the protein binding of uremic toxins in patients with different stages of CKD and during a hemodialysis session. The observed change in protein binding in CKD patients with advanced CKD stages might be due to post-translational modifications of proteins, characteristic for CKD progression. The observed results in HD patients explain why protein-bound solutes are difficult to remove by dialysis: a combination of the fact that (i) only the free fraction can pass the filter and (ii) the equilibrium, as it was pre-dialysis, cannot be restored during the dialysis session, as it is continuously disturbed. This can be explained by the kinetics of these protein-bound uremic toxins: once the free fraction is removed, the equilibrium with the bound fraction and the extra vascular space is disturbed, causing a potential release of the bound fraction, respectively inflow from the extravascular space [20].

## Supplementary Materials

Supplementary materials can be accessed at: http://www.mdpi.com/2072-6651/7/10/3933/s1.
