# Supplementary material for: Exploring Protein Binding of Uremic Toxins in Patients with Different Stages of Chronic Kidney Disease and during Hemodialysis
Source: Toxins (Basel). 2015 Sep 28;7(10):3933–46. doi: 10.3390/toxins7103933 (PMC4626712; doi:10.3390/toxins7103933)
Supplement: Supplementary file 1 [file toxins-07-03933-s001.pdf]

Supplementary Information

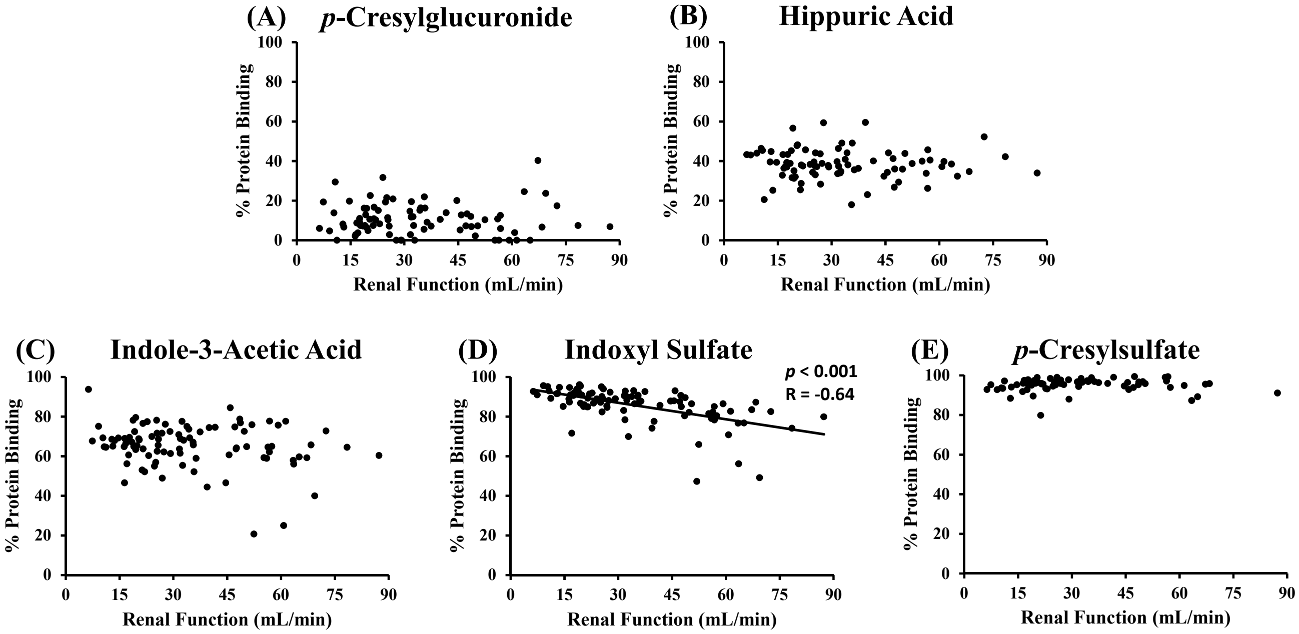

Figure S1. Percentage protein binding (%PB) *versus* renal function of CKD patients for: (**A**) *p*-cresylglucuronide; (**B**) hippuric acid; (**C**) indole-3-acetic acid; (**D**) indoxyl sulfate (with Spearman *p*- and *R*-value); and (**E**) *p*-cresylsulfate.

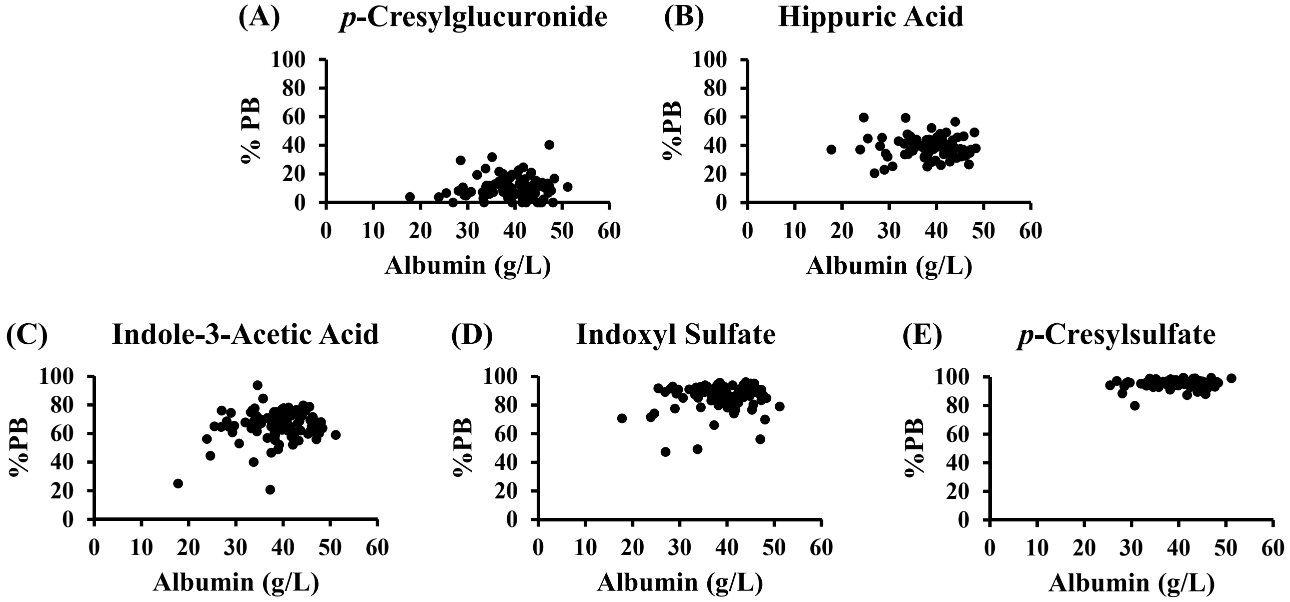

Figure S2. Protein binding *versus* albumin concentration of CKD patients for: (**A**) *p*-cresylglucuronide; (**B**) hippuric acid; (**C**) indole-3-acetic acid; (**D**) indoxyl sulfate; and (**E**) *p*-cresylsulfate.

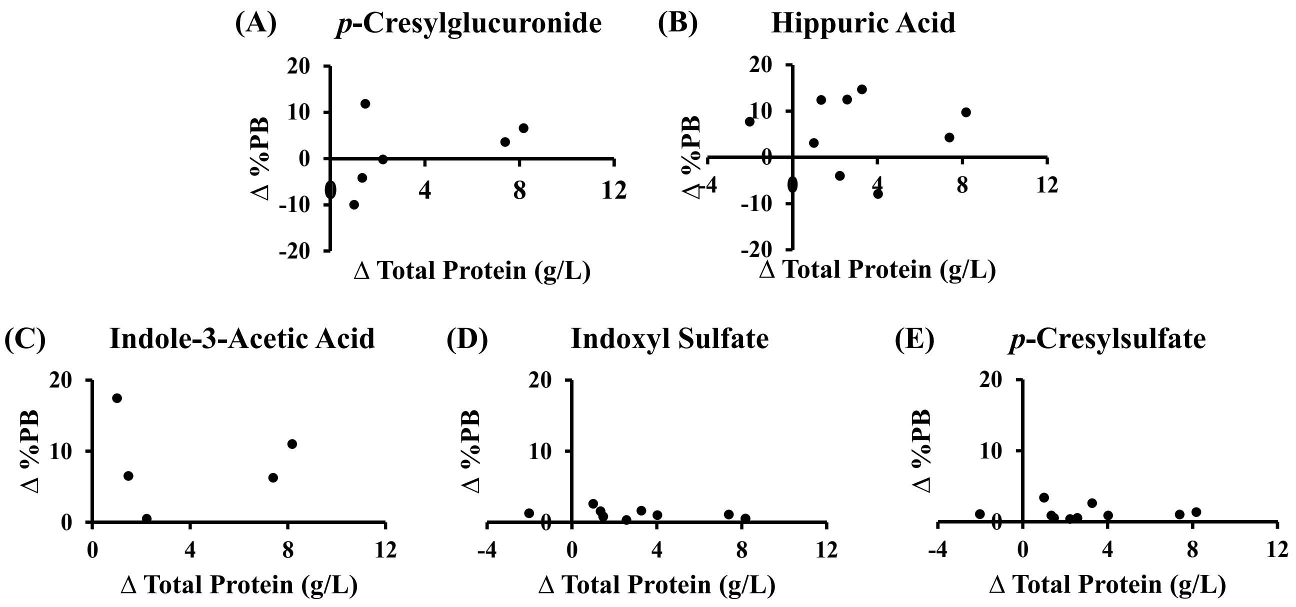

Figure S3. Change (Δ) in protein binding *versus* change in total protein concentration of HD patients at the inlet and the outlet of the dialyzer, after 120 min of dialysis for: (**A**) *p*-cresylglucuronide; (**B**) hippuric acid; (**C**) indole-3-acetic acid; (**D**) indoxyl sulfate; and (**E**) *p*-cresylsulfate.
